# Non-invasive evaluation of facial crestal bone with ultrasonography

**DOI:** 10.1371/journal.pone.0171237

**Published:** 2017-02-08

**Authors:** Hsun-Liang Chan, Khaled Sinjab, Ming-Pang Chung, Yi-Chen Chiang, Hom-Lay Wang, William V. Giannobile, Oliver D. Kripfgans

**Affiliations:** 1 Department of Periodontics and Oral Medicine, University of Michigan School of Dentistry, Ann Arbor, MI, United States of America; 2 Department of Biomedical Engineering, College of Engineering, Ann Arbor, MI, United States of America; 3 Department of Radiology, University of Michigan Medical School, Ann Arbor, MI, United States of America; Virginia Commonwealth University, UNITED STATES

## Abstract

**Purpose:**

Facial crestal bone level and dimension determine function and esthetics of dentition and dental implants. We have previously demonstrated that ultrasound can identify bony and soft tissue structures in the oral cavity. The aim of this study is to evaluate the accuracy of using ultrasound to measure facial crestal bone level and thickness.

**Materials and methods:**

A commercially available medical ultrasound scanner, paired with a 14 MHz imaging probe was used to scan dental and periodontal tissues at the mid-facial site of each tooth on 6 fresh cadavers. The alveolar crest level in relation to the cemento-enamel junction and its thickness on ultrasound images were measured and compared to those on cone-beam computed tomography (CBCT) scans and/or direct measurements on a total of 144 teeth.

**Results:**

The mean crestal bone level measured by means of ultrasound, CBCT and direct measures was 2.66 ± 0.86 mm, 2.51 ± 0.82 mm, and 2.71 ± 1.04 mm, respectively. The mean crestal bone thickness was 0.71 ± 0.44 mm and 0.74 ± 0.34 mm, measured by means of ultrasound and CBCT, respectively. The correlations of the ultrasound readings to the other two methods were between 0.78 and 0.88. The mean absolute differences in crestal bone height and thickness between ultrasound and CBCT were 0.09 mm (-1.20 to 1.00 mm, p = 0.06) and 0.03 mm (-0.48 to 0.54 mm, p = 0.03), respectively.

**Conclusion:**

Ultrasound was as accurate in determining alveolar bone level and its thickness as CBCT and direct measurements. Clinical trials will be required to further validate this non-ionizing and non-invasive method for determining facial crestal bone position and dimension.

## Introduction

The thickness and level of the facial alveolar bone are important prognostic parameters for functional and esthetic outcomes of periodontal and dental implant therapy. For instance, gingival recession is commonly accompanied with thin facial bone and/or bony dehiscence [1]. Sockets with a thin buccal plate have approximately two times more horizontal and vertical bone resorption after immediate implant placement [2]. Extraction sockets with thin buccal bone (≤1 mm), a majority (71%) have significant horizontal and vertical buccal bone resorption during the healing period. Therefore, it is critical to diagnose the dimension and location of the facial plate before performing these procedures.

Clinical evaluation of facial bone is an invasive procedure that requires flap elevation or sounding under local anesthesia. Therefore, advanced imaging techniques such as three-dimensional cone-beam computed tomography (CBCT) have been used to study facial bone thickness [3–5]. However, its accuracy at sites with very thin facial bone, high radiation dose, and significant cost limits its use in daily clinical practice. Non-ionizing, real-time, and cost-effective ultrasound imaging has been used extensively in quantitative medical diagnostics, such as, in evaluating the developing fetal dimensions [6]. In dentistry, ultrasound imaging is mainly used as a research tool for evaluating soft tissue lesions [7–11] and gingival thickness [12, 13]. Our recent proof-of-principle study [14] demonstrated that ultrasound imaging could provide cross-sectional periodontal surface images with unprecedented soft tissue contrast and well-delineated hard tissue surface topography. Therefore, this study aims to investigate the accuracy of sonography in measuring facial crestal bone level and thickness on different tooth types with a larger sample size.

## Materials and methods

### Specimen acquisition and CBCT scans

This was a non-regulated study, as determined by the University of Michigan Institutional Review Board (Study ID: HUM00107975). Six fresh cadaveric heads, kept frozen at -20°C and thawed at the initiation of the experiment, were scanned using a CBCT scanner (3D Accuitomo 170, JMorita, Japan), with scanning parameters of 120 kVp, 18.66 mAs, scan time of 20 seconds, and resolution of 80 μm. A plastic cheek retractor and cotton rolls were used to separate facial mucosae from gingiva/alveolar mucosae. The captured CBCT scans were three-dimensionally reconstructed with the built-in software, saved in DICOM format, and subsequently exported into a commercially available implant planning software (Invivo5, Anatomage Dental, San Jose, CA, USA).

### Ultrasound scanning

A single periodontist (HC) performed the ultrasound scanning while another examiner (OK), specialized in ultrasound imaging, controlled the scanner. A clinical ultrasound scanner (ZS3, Zonare, Mountain View CA, USA) paired with a 14 MHz intraoperative imaging probe (L14-5sp) was used. The scanning set-up and procedures have been previously described [14]. Briefly, a spatial compounding function was selected to acquire smoother bone and tooth edges. Acoustic coupling was achieved by applying ultrasound gel and the use of gel-based stand-off-pads. Each tooth was scanned at the mid-facial surface with the transducer placed approximately in line with the long axis of the tooth. Several ultrasound scans with minute differences in the faciolingual scan plane in relation to the teeth were acquired and saved in DICOM format for each tooth.

### Quantitative analysis

Two linear measurements were made at the mid-facial site of each tooth on ultrasound images by one calibrated examiner (HC), and compared to those on CT images measured by another calibrated examiner (YC) and/or direct measurements by the 3^rd^ calibrated examiner (MC): (1) crestal bone level, i.e., the vertical distance between the alveolar crest and the cemento-enamel junction (CEJ), and (2) crestal bone thickness, i.e., the horizontal distance between the outer surface of the alveolar crest and the root dentin surface. Before the experiment, two examiners (YC and MC) were calibrated to the standard examiner (HC) by measuring 10 sites in one cadaver head until a correlation coefficient of at least 0.9 was achieved. Intra-examiner calibrations were performed through measuring 10 sites in one cadaver head repeatedly approximately once every day in order to achieve a correlation coefficient of at least 0.9. After ultrasound and CT scans were performed, a full thickness facial flap was elevated to reveal the facial alveolar bone on each cadaver for direct measurements. Direct measurements were made with a calibrated periodontal probe (University of North Carolina (UNC) Probe, Hu-Friedy, Chicago, IL, USA) accurate to 1 mm. Measurements on ultrasound and CBCT images were made with the built-in digital caliper in the software in millimeter. The mean differences of the two parameters between the ultrasound, CBCT, and direct assessment were calculated. The correlations and agreement between measurements from ultrasound, CBCT and direct assessment were evaluated with the Pearson’s correlation coefficient test and the Bland-Altman analysis, respectively. The significant level was set at 0.05 for all statistical analysis.

## Results

**Fig 1:**
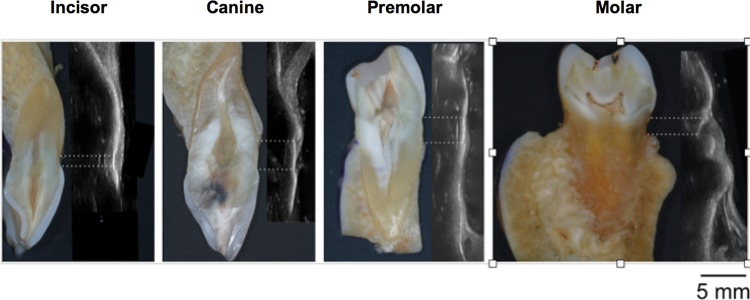
Demonstrations of ultrasound images for different tooth types in relation to the ground sections of the respective teeth. The two dashed lines represent the CEJ and alveolar bone crest level, respectively.

**Fig 2:**
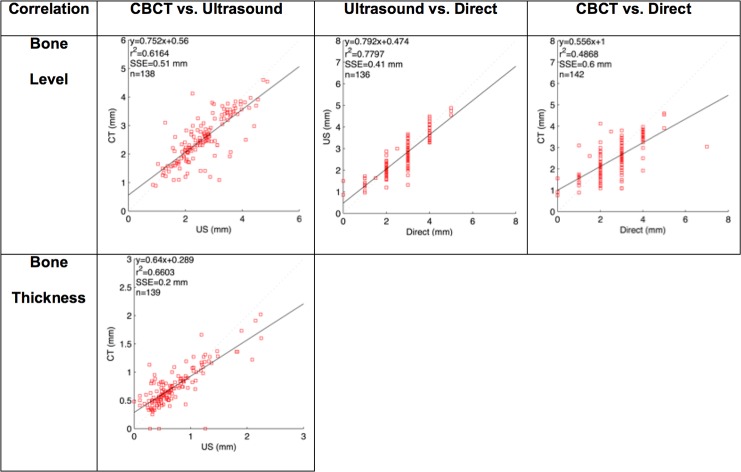
Correlations of bone level and thickness readings between 3 estimation methods.

**Fig 3:**
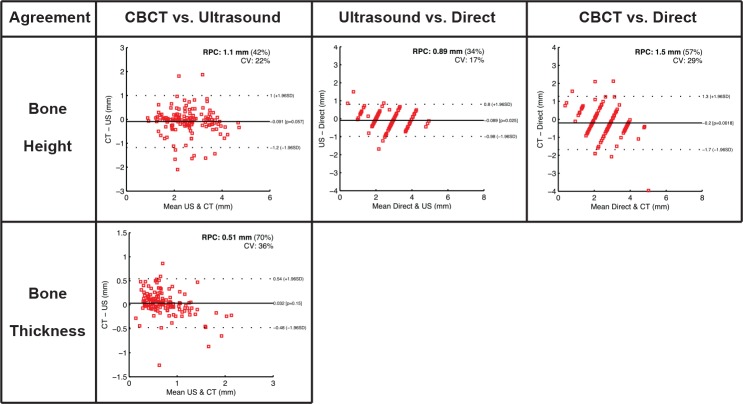
Agreement of bone level and thickness readings between 3 estimation methods.

Six cadaver heads, 3 males and 3 females with a mean age of 75.2 (range: 66 to 89) years were studied. A total number of 144 teeth were measured with the CBCT method, including 67 anterior teeth, 37 premolars and 40 molars in both maxillae and mandiblae. Of those teeth, five teeth (three 2^nd^ molars and 2 canines) were excluded for ultrasound due to suboptimal image quality (Table 1). Fig 1 illustrates the ultrasound images of different tooth types. The landmarks, including the CEJ and facial alveolar crest were clearly demarcated and correlated with those on the cadaver specimens. Additionally, the tooth and bone surface contours as well as gingiva/mucosa were identifiable. The mean crestal bone level measured from ultrasound images, CBCT images and cadavers directly was 2.66 ± 0.86 mm, 2.51 ± 0.82 mm, and 2.71 ± 1.04 mm, respectively (Table 2). Likewise, the mean crestal bone thickness was 0.71 ± 0.44 mm and 0.74 ± 0.34 mm, measured from ultrasound and CBCT images, respectively (Table 1). Statistically significant correlations were found for bone height readings between ultrasound and direct methods (r = 0.88, p<0.001), between ultrasound and CBCT methods (r = 0.78, p<0.001), and direct and CBCT methods (r = 0.70, p<0.001) (Table 3 and Fig 2). A statistically significant correlation was also found for bone thickness measurements obtained between ultrasound imaging and CBCT scans (r = 0.81, p<0.001) (Table 3 and Fig 2). The mean absolute difference (95% CI) in bone level between ultrasound and direct, ultrasound and CBCT, CBCT and direct was 0.09 mm (-0.98 to 0.80 mm, p = 0.03), 0.09 mm (-1.20 to 1.00 mm, p = 0.06), 0.20 mm (-1.70 to 1.30 mm, p = 0.018), respectively (Table 3 and Fig 3). The mean absolute difference (95% CI) in bone thickness between ultrasound and CBCT was 0.03 mm (-0.48 to 0.54 mm, p = 0.03) (Table 3 and Fig 3).

**Table 1:** Distributions of samples in relation to tooth type and bone locations among 3 estimation methods.

Methods	Tooth type / Location	Maxilla	Mandible	Subtotal
**Ultrasound**	Anterior	34	31	65
	Premolar	17	20	37
	Molar	18	19	37
	Subtotal	69	70	**139**
**Direct measure**	Anterior	34	32	66
	Premolar	17	20	37
	Molar	20	20	40
	Subtotal	71	72	**143**
**CBCT**	Anterior	35	32	67
	Premolar	17	20	37
	Molar	20	20	40
	Subtotal	72	72	**144**

Key: CBCT: cone-beam computed tomography. Five teeth were excluded in Ultrasound group due to inadequate image quality. One tooth was excluded from direct measurement group due to facial plate fracture during specimen preparation.

**Table 2:** Comparisons of the mean (SD) alveolar bone level and thickness between 3 estimation methods.

Mean (SD) (mm)	Tooth type	Ultrasound	Direct measure	CBCT
**Bone level**	Anterior	2.81 (0.81)	2.96 (1.08)	2.72 (0.77)
	Premolar	2.54 (0.81)	2.66 (0.86)	2.44 (0.83)
	Molar	2.37 (0.93)	2.35 (1.02)	2.23 (0.83)
	All teeth	2.66 (0.86)	2.71 (1.04)	2.51 (0.82)
**Bone thickness**	Anterior	0.62 (0.42)	NA	0.68 (0.34)
	Premolar	0.64 (0.35)		0.73 (0.25)
	Molar	0.93 (0.49)		0.84 (0.39)
	All teeth	0.71 (0.44)		0.74 (0.34)

NA: not measured.

**Table 3:** Results of correlation coefficient test and agreement analysis between the 3 estimation methods.

Parameters	Methods	Correlation (R)	Mean mm absolute difference (P value)	95% CI (mm)
**Bone level**	US vs. Direct	0.88	0.09 (0.03)	-0.98 to 0.80	
	US vs. CBCT	0.78	0.09 (0.06)	-1.20 to 1.00	
	CBCT vs. Direct	0.70	0.20 (0.018)	-1.70 to 1.30	
**Bone thickness**	US vs. CBCT	0.81	0.03 (0.15)	-0.48 to 0.54	

US: ultrasound.

## Discussion

Tissue biotype is considered an important determinant of treatment outcomes in the management of periodontal diseases [15, 16], in bone regenerative procedures [17], and in implant therapy [18–20]. While significant efforts have been placed in developing methods to evaluate soft tissue biotypes [21, 22], it is undeniable that the underlying hard tissue influences the overlying tissue biotype [23]. It has been suggested that a moderate correlation exists between facial gingival thickness and underlying bone thickness [4]. Ultrasound has proven a suitable tool for evaluating soft tissue, e.g., gingival/mucosal thickness [12, 13, 24]. Although ultrasound has been used for evaluating bone surface topography in orthopedics, it is the first time to apply this technology to quantify the alveolar bone dimension and correlate the readings with radiographic and direct measurements on human cadavers. Further improvement of ultrasound accuracy by increasing spatial resolution could make this novel technique a clinical reality.

Cone beam computed tomography has been used to evaluate alveolar bone dimensions using cadaveric specimens [5]. The authors reported that the mean absolute differences between CBCT and direct assessments were 0.30 mm for buccal bone height and 0.13 mm for buccal bone thickness with 95% limits of agreement of −0.77 to 0.81 mm, and −0.32 to 0.38 mm, respectively. The mean facial bone thickness of maxillary teeth was found to be between 0.5 and 0.7 mm [3]. Additionally, in approximately 90% of the examined teeth, the facial wall thickness is less than 1 mm. Central incisors and canines have less favorable conditions, with only 4.6% and 8.6% having a thick bony wall (equal or more than 1 mm), respectively. Another study [25] showed that the median distance from the CEJ to the facial alveolar bone crest is 2.79 mm and the median facial alveolar bone thickness for the maxillary anterior teeth is 0.88 mm. Likewise, a CBCT study with 300 patients showed that 80% of anterior teeth have less than 1 mm of facial bone thickness [26]. A recent study also demonstrated that the maxillary anterior teeth have thin facial bone thickness (mean thickness of 0.76 mm), with thick biotype having thicker bone, although the mean difference did not achieve statistical significance [27].

A limitation of this study is that the measurement sites among the 3 assessment methods were non-standardized with reproducible landmarks. Every effort was made to generate readings at the mid-facial site of each tooth. Second, locating the CEJ might be challenging for some teeth, especially when tooth abrasion had occurred. The inability to identify the CEJ reproducibly may have negatively influenced the agreement on measurements of alveolar crest level. Third, bone thickness measurements are only reliable at the junction of the tooth root and bone surface on ultrasound images because sound with the frequency used in this study cannot transmit through the bony housing.

This non-ionizing, non-invasive and real-time device can have an extended application on evaluating and monitoring facial bone around implants. The adaptation of using this device to study facial bone quantity around implants will significantly enhance our understanding of soft-hard tissue interactions and implant esthetics in this critical anatomical region.

This human cadaver study will aid in the development of live human studies. For use in live humans, probes with a higher resolution and miniatured size are required. The currently used 14 MHz probe can reach a spatial resolution of approximately 100 μm. Higher frequency can increase the resolution and possibly result in a higher accuracy for imaging facial bone topography. A tooth sized probe should be an optimal size for intraoral use.

## Conclusions

Facial alveolar bone dimension was assessed with a commercially available medical ultrasound scanner and compared to those measured directly on specimens and by cone beam computed tomography. Strong correlations (r>0.80) and agreements (mean differences< 0.1 mm) of ultrasound readings to the other two methods hold promise for clinical use of ultrasound for evaluating crestal bone level as well as the thickness.
